# A 6-year cohort study of the associations of body mass index, waist circumference, and waist-hip ratio with cognitive impairment in Chinese elderly

**DOI:** 10.3389/fpsyg.2026.1833217

**Published:** 2026-07-03

**Authors:** Qianle Zhou, Xiaoyi Zhang, Tao Zhang, Le Xu, Xue Gu, Junfen Lin, JunHao Ma, Fudong Li

**Affiliations:** 1 School of Public Health, Hangzhou Medical College, Hangzhou, China; 2 Tongxiang Maternity and Child Health Care Hospital, Tongxiang, Zhejiang, China; 3 Zhejiang Provincial Center for Disease Control and Prevention, Zhejiang, Hangzhou, China; 4 Zhejiang Key Lab of Vaccine, Infectious Disease Prevention and Control, Zhejiang, Hangzhou, China

**Keywords:** BMI, cognitive impairment, older people, WC, WHR

## Abstract

**Objectives:**

Current findings from cohort studies examining the association between obesity and cognitive impairment are inconsistent, particularly with regard to central obesity. This study aimed to investigate the prospective associations of Body Mass Index (BMI), Waist Circumference (WC), and Waist-Hip Ratio (WHR) with the risk of cognitive impairment among Chinese elderly.

**Methods:**

We used data from the Zhejiang Healthy Aging Cohort Study (ZHACS), which included 8,443 participants. BMI, WC, and WHR were assessed using a baseline questionnaire that was initiated in 2014. Cognitive function was evaluated using the Mini-Mental State Examination (MMSE) at baseline and three waves of follow-up (2015, 2016, and 2019–2021), with cognitive impairment based on thresholds tailored to participants’ educational backgrounds. Log-binomial regression models adjusted for numerous covariates were used to examine the associations and calculate relative risks (RRs).

**Results:**

After 6 years of follow-up, 3,006 participants (35.60%) showed cognitive impairment at least once, according to the MMSE. BMI-defined underweight was positively associated with cognitive impairment (RR = 1.15; 95%CI: 1.05–1.27). Conversely, inverse associations were identified for central obesity defined by WC and WHR, with estimated effect sizes of 14% (RR = 0.86; 95%CI: 0.81–0.91) and 9% (RR = 0.91; 95%CI: 0.86–0.97), respectively. Participants with both BMI-defined overweight/obesity and WC/WHR-defined central obesity had a significant inverse association with cognitive impairment, whereas those with both BMI-defined underweight and WC/WHR-defined central obesity did not. Stratified analyses indicated that the association of BMI-defined underweight and WHR-defined central obesity with cognitive impairment was more evident among women participants and individuals aged ≥70 years.

**Conclusion:**

Among Chinese older adults, BMI-defined underweight was significantly positively associated with cognitive impairment, whereas WC-defined and WHR-defined central obesity was inversely associated with cognitive impairment, particularly among older adults (≥70 years) and women.

## 1 Introduction

Against the backdrop of an accelerating global population aging, cognitive impairment has emerged as a core health issue affecting the elderly (Campos et al., 2024). It is not only a significant manifestation of aging but also the primary symptom of dementia (Ushakova et al., 2025). Among these, Alzheimer’s disease (AD) accounts for 60–80% of all dementia cases, making it the most common cause (Alzheimer’s Association, 2024). This disease poses an increasingly severe challenge to global public health. Statistics indicate that approximately 56.9 million people worldwide currently suffer from Alzheimer’s disease and other types of dementia (ADRD), with this figure projected to surge to approximately 152.8 million by 2050 (Qin et al., 2025). The situation in China is as severe as that in India. Projections indicate that by 2050, the number of elderly individuals with cognitive impairment in China may peak at approximately 44.59 million to 51.41 million (Cheng et al., 2025). The burden of cognitive impairment is both comprehensive and severe. Economically, the total societal cost of dementia worldwide reached approximately $1.3 trillion in 2019 (Li et al., 2025). For families of patients, cognitive decline directly impairs older adults’ ability to perform complex daily activities, such as managing finances, preparing meals, and taking medications on schedule, severely affecting their independence and quality of life. Therefore, identifying modifiable correlates of cognitive impairment and exploring effective early intervention strategies have become urgent needs in addressing the health challenges of an aging society.

Obesity is a controllable contributor to cognitive disorders such as dementia (Dye et al., 2017). Investigators commonly assess obesity using metrics such as Body Mass Index (BMI), Waist Circumference (WC), and Waist-Hip Ratio (WHR). However, despite the availability of multiple assessment methods, the association between obesity, particularly central obesity, and cognitive disorders remains unclear. Some studies have indicated that obesity may accelerate cognitive decline, leading to cognitive impairment or dementia (Rubin et al., 2019; Prickett et al., 2015; Sanderlin et al., 2017; Souza-Lima et al., 2023). However, other studies have also indicated that being underweight may have a significant positive association with cognitive impairment, while obesity has a significant inverse association with cognitive decline and dementia in older adults (Qu et al., 2020; Gu et al., 2025; Aiken-Morgan et al., 2020; Miladitiya et al., 2023; Momtaz et al., 2018). Second, even for central obesity indicators such as WC and WHR, findings across different studies remain inconsistent, and no consensus has been reached. Previous studies have found that a higher WC/WHR is associated with better cognitive function (Wu et al., 2025; Liang et al., 2022; Zhao et al., 2024). Additional studies have indicated that a higher WC/WHR is associated with accelerated cognitive decline, whereas BMI is unrelated to the rate of decline (Kuk and L., 2010; West et al., 2017; Sloots, 2024). In addition, there are significant differences between men and women in terms of obesity and weight change (Link and Reue, 2017; Jiang et al., 2022). Another study found that (Tang et al., 2021) the risk of WC on cognitive impairment was only significant in individuals aged ≥65 years. Therefore, the association between WC/WHR and cognitive impairment may vary depending on factors such as sex and age. Finally, it is worth noting that to date, few studies have combined BMI with indicators such as WC/WHR for analysis within a single cohort. A study found that a comprehensive analysis of BMI, WC, and WHR can more accurately identify the risk of cognitive impairment, thereby addressing the limitations of using BMI alone to reflect fat distribution patterns (Gu et al., 2018). Only Liu et al. (2019) examined the combined effects of BMI and WC/WHR. However, the BMI grouping failed to include the “underweight” category, which represents an equally important risk group in the elderly population. Furthermore, the analysis did not account for the influence of gender, and the cross-sectional survey design has inherent limitations.

Given the limited number of cohort studies currently examining the association between BMI, WC, WHR, and cognitive impairment while accounting for sex and age differences, this study used data from a large-scale cohort study in Eastern China. We introduced combined indicators of BMI and WC/WHR to investigate their role across different sex and age groups, thereby thoroughly exploring the relationship between BMI, WC, WHR, and cognitive impairment in the elderly. Our objective was to gather sufficient evidence to address the gaps in existing research.

## 2 Methods

### 2.1 Participants

The data for this study were obtained from the Zhejiang Healthy Aging Cohort Study (ZHACS; Gu et al., 2026), a large-scale, community-based longitudinal investigation that monitors health trends in elderly Chinese individuals. Led by the Zhejiang Provincial Center for Disease Control and Prevention, the study employed a multistage sampling method: first, seven counties were selected from the province’s 90 counties, and then one township or urban subdistrict was chosen per county. Researchers randomly selected 2–3 villages or residential communities within each location and extended invitations to all qualifying residents. The eligibility criteria required participants to be permanent community residents aged 60 years or older. This study identified 13,955 qualified participants from the selected region.

The study began its baseline survey in 2014, involving 10,911 elderly participants. Subsequently, the researchers screened and excluded 10 duplicate records, ultimately reducing the sample size to 10,901 participants who completed the questionnaire. Follow-up assessments were conducted in three rounds: 2015, 2016, and 2019–2021. However, budgetary restraints forced the second round (2016) to focus on only six counties, while the first and third rounds encompassed all the seven counties.

#### 2.1.1 Selection of participants

**Figure 1:**
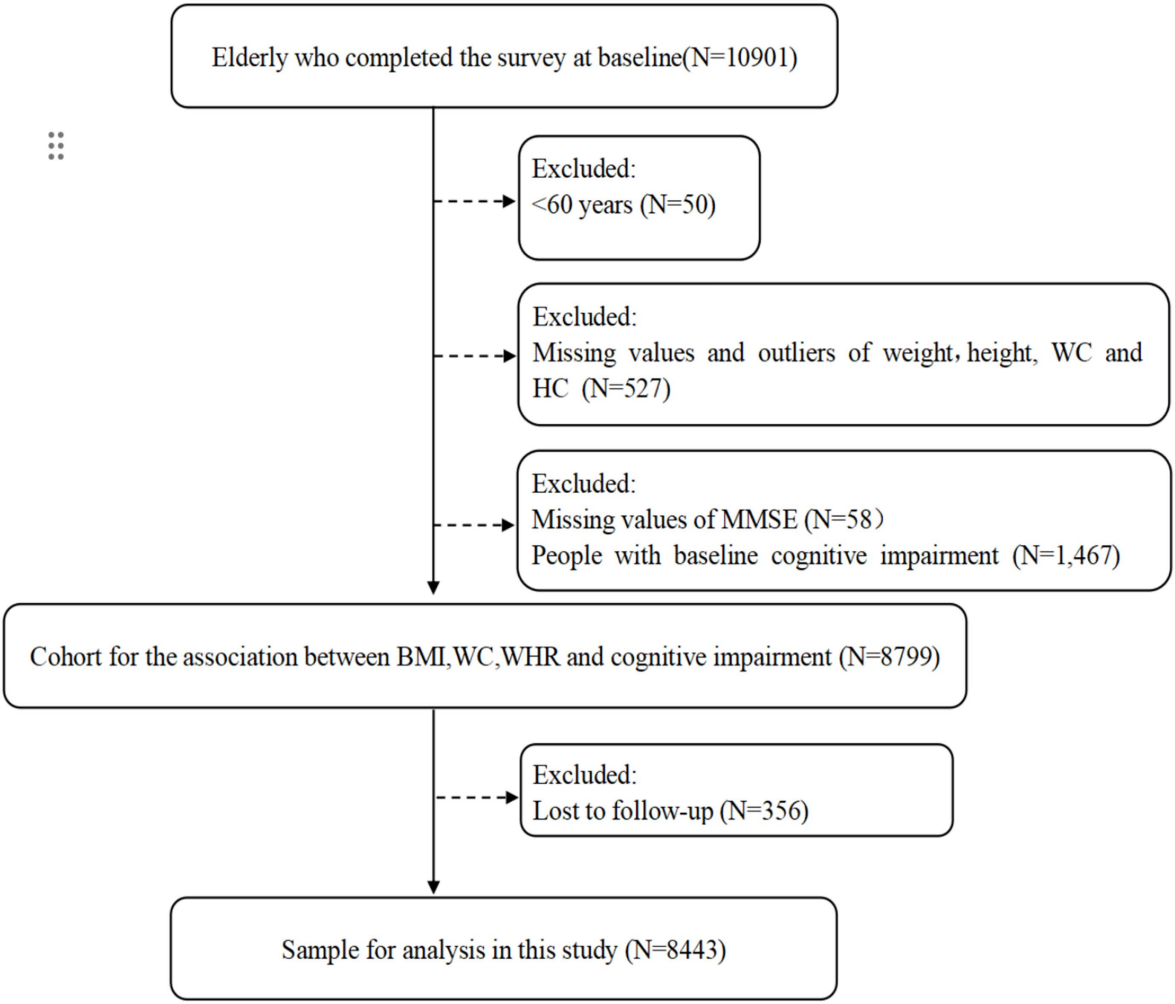
Diagram illustrating the process of selecting participants for assessing associations between BMI, WC, WHR, and cognitive impairment.

Of the 10,901 participants enrolled in the baseline survey of the Zhejiang Healthy Aging Cohort Study (ZHACS), 8,443 were included in the final analysis. Exclusions were made for the following reasons: age <60 years (*n* = 50), missing cognitive assessments (*n* = 58), missing anthropometric data (weight, height, WC, and HC; *n* = 527), and presence of cognitive impairment at baseline (*n* = 1,467). Furthermore, the analysis excluded 356 participants who were lost to follow-up. The missing data for all study covariates were low and randomly distributed. Participants with missing anthropometric, cognitive assessment, or covariate data were excluded, and a complete case analysis was performed without additional imputation (Figure 1).

#### 2.1.2 Baseline characteristics of the study participants

This study included 8,443 seniors (4,126 men and 4,316 women) aged 60–95 years, with a median age of 67 years. In terms of education level, 3,878 participants (45.93%) were illiterate, 3,771 (44.66%) had completed primary school, and 794 (9.40%) had a junior high school education or above. Of the 8,443 participants, 3,006 (35.60%) were defined as having cognitive impairment based on MMSE scores. Based on BMI, 422 individuals (5.00%) were classified as underweight and 5,464 (64.72%) as normal weight. Additionally, 1,796 participants (21.27%) were categorized as overweight and 761 (9.01%) as obese. The average WC was 83.27 cm (±9.28), with 4,081 participants (48.34%) identified as having central obesity based on WC. The mean WHR was 0.90 (±0.07), and 2,657 participants (31.47%) were centrally obese based on their WHR (Table 1).

**Table 1:** Overview of baseline characteristics by cognitive impairment status.

Variables	Total (*n* = 8,443)	No cognitive impairment (*n* = 5,437)	Cognitive impairment (*n* = 3,006)	*p*-value
Sex				<0.001
Men	4,126 (48.87)	2,814 (51.77)	1,312 (43.65)	
Women	4,316 (51.13)	2,622 (48.23)	1,694 (56.35)	
Ethnicity				0.83
Han Chinese	8,242 (97.62)	5,309 (97.65)	2,933 (97.57)	
Other	201 (2.38)	128 (2.35)	73 (2.43)	
Age groups				<0.001
60–64 years	2,895 (34.29)	2,110 (38.81)	785 (26.11)	
65–74 years	3,758 (44.51)	2,441 (44.90)	1,317 (43.81)	
75–84 years	1,615 (19.13)	807 (14.84)	808 (26.88)	
≥85 years	175 (2.07)	79 (1.45)	96 (3.19)	
Education				<0.001
Illiteracy	3,878 (45.93)	2,352 (43.26)	1,526 (50.77)	
Primary school	3,771 (44.66)	2,571 (47.29)	1,200 (39.92)	
Junior high school and above	794 (9.40)	514 (9.45)	280 (9.31)	
Work status				<0.001
Working	3,366 (39.90)	2,358 (43.39)	1,008 (33.58)	
Retired	4,598 (54.50)	2,798 (51.49)	1,800 (59.96)	
Never	472 (5.60)	278 (5.12)	194 (6.46)	
Economic status				<0.001
Low	752 (8.91)	417 (7.67)	335 (11.14)	
Medium	6,895 (81.67)	4,454 (81.92)	2,441 (81.20)	
High	796 (9.43)	566 (10.41)	230 (7.65)	
Marital status				<0.001
Married	6,614 (78.34)	4,425 (81.39)	2,189 (72.82)	
Widowed	1,664 (19.71)	912 (16.77)	752 (25.02)	
Unmarried/divorced	165 (1.95)	100 (1.84)	65 (2.16)	
Smoking				<0.001
Never	5,855 (69.35)	3,693 (67.92)	2,162 (71.92)	
Current	1,761 (20.86)	1,169 (21.50)	592 (19.69)	
Former	827 (9.80)	575 (10.58)	252 (8.38)	
Drinking				<0.001
Never	5,537 (65.58)	3,495 (64.28)	2,042 (67.93)	
Current	2,246 (26.60)	1,527 (28.09)	719 (23.92)	
Former	660 (7.82)	415 (7.63)	245 (8.15)	
Exercise				<0.001
Yes	1,689 (20.00)	1,202 (22.11)	487 (16.20)	
No	6,754 (80.00)	4,235 (77.89)	2,519 (83.80)	
Hypertension				0.007
Yes	3,769 (44.64)	2,368 (43.55)	1,401 (46.61)	
No	4,674 (55.36)	3,069 (56.45)	1,605 (53.39)	
Diabetes				0.037
Yes	762 (9.03)	517 (9.51)	245 (8.15)	
No	7,681 (90.97)	4,920 (90.49)	2,761 (91.85)	
Stroke				0.002
Yes	209 (2.48)	113 (2.08)	96 (3.19)	
No	8,234 (97.52)	5,324 (97.92)	2,910 (96.81)	
Depressive symptoms				0.174
Yes	649 (7.69)	402 (7.39)	247 (8.22)	
No	7,794 (92.31)	5,035 (92.61)	2,759 (91.78)	
BMI (kg/m^2^)	23.43 ± 3.28	23.61 ± 3.23	23.11 ± 3.35	<0.001
BMI groups				<0.001
Underweight (<18.5)	422 (5.00)	216 (3.97)	206 (6.85)	
Normal (18.5–23.9)	5,464 (64.72)	3,503 (64.43)	1,961 (65.24)	
Overweight (24.0–27.9)	1,796 (21.27)	1,204 (22.14)	592 (19.69)	
Obesity (≥28.0)	761 (9.01)	514 (9.45)	247 (8.22)	
WC (cm)	83.27 ± 9.28	83.81 ± 9.26	82.28 ± 9.24	<0.001
WC groups				<0.001
Normal	4,362 (51.66)	2,892 (53.19)	1,470 (48.90)	
Central obesity	4,081 (48.34)	2,545 (46.81)	1,536 (51.10)	
WHR	0.90 ± 0.07	0.90 ± 0.06	0.90 ± 0.07	<0.001
WHR groups				0.493
Normal	5,786 (68.53)	3,740 (68.79)	2,046 (68.06)	
Central obesity	2,657 (31.47)	1,697 (31.21)	960 (31.94)	

BMI = body mass index, WC = waist circumference, WHR = waist-to-hip ratio.

### 2.2 Tests administered and body measurements

#### 2.2.1 Questionnaire

The questionnaire covered basic demographic information, family status, reproductive history, medical history, behavioral habits, dietary habits, history of trauma, depressive symptoms, activities of daily living, and cognitive function.

#### 2.2.2 Assessment of cognitive impairment

Cognitive impairment was identified using the Chinese version of the Mini-Mental State Examination (MMSE; Hu, M. et al., 2021) in the baseline questionnaire and each follow-up survey.

The Mini-Mental State Examination (MMSE), which consists of 30 standardized items, assesses orientation to time and place, attention and calculation skills, recent and remote memory, and language function; the total score ranges from 0 to 30 points. Education-specific MMSE thresholds were adopted in this study, as these stratified cutoffs have been wellvalidated and culturally adapted for older Chinese adults (Li et al., 2016; Cui et al., 2011). Cognitive impairment thresholds vary according to education level: ≤17 (illiterate), ≤20 (primary school), and ≤24 (junior high or above; Gao et al., 2021). Three rounds of MMSE assessments were conducted during the follow-up in 2015, 2016, and 2019–2021. For individuals who remained cognitively intact at baseline, incident cognitive impairment was defined as meeting the MMSE threshold for cognitive impairment at first occurrence during follow-up wave. All MMSE assessments were administered using the same procedure across all follow-up waves.

#### 2.2.3 Anthropometric measurements of adiposity

In the baseline survey, the participants’ heights (cm) and weights (kg) were measured using an electronic scale and height gauge, respectively, without the use of footwear or bulky clothing. To accurately measure waist circumference, the participants were instructed to maintain normal breathing while the examiner placed the measuring tape precisely between the top of the hip bone and the bottom edge of the ribs. For hip circumference, the measurement was taken around the fullest part of the buttocks, using the front of the pubic bone as the key landmark (Gunstad et al., 2010; Ma et al., 2021). BMI was calculated as weight in kilograms divided by the square of height in meters. WHR was calculated by dividing the waist circumference by the hip circumference.

Based on the criteria for Chinese adults (Hu et al., 2018), BMI (kg/m^2^) was categorized into four groups: underweight (<18.5 kg/m^2^), normal weight (18.5–23.9 kg/m^2^), overweight (24–27.9 kg/m^2^), and obese (≥28 kg/m^2^). Central obesity was determined using WC thresholds of ≥90 cm for men and ≥85 cm for women (Notice of the General Office of the National Health Commission on the Issuance of 2023 National Food Safety Standards Project Approval Plan, 2023; Sun et al., 2024). WHR values below 0.9 for men and 0.85 for women were considered normal, whereas higher values indicated central obesity (Wang, n.d.).

### 2.3 Procedures

Prior to the formal survey, all interviewers received standardized professional training to ensure consistent data collection. All assessments and questionnaire surveys were completed through one-on-one face-to-face interviews, using dedicated interview software on electronic tablets. Data collection was generally conducted in the morning or early afternoon to avoid participant fatigue. A fixed and unified assessment sequence was strictly implemented across all participants: anthropometric measurements were collected first, followed by the administration of cognitive function tests. No formal scheduled breaks were set during the assessment procedure, while participants were permitted to take voluntary short rests if they felt fatigued.

### 2.4 Statistical procedures

#### 2.4.1 Covariates

This analysis was adjusted for demographic, socioeconomic, and health-related confounders: age (60–64, 65–74, 75–84, or ≥85 years), sex (men, women), ethnicity (Han Chinese, other), education (illiteracy, primary school, junior high school and above), marital status (married, widowed, unmarried/divorced), economic status (low, medium, high), work status (working, retired, never worked), exercise (activities carried out to sustain or improve health and fitness in one’s spare time, yes, no), smoking (never, current, former), drinking (never, current, former), hypertension (yes, no), diabetes (yes, no), stroke (yes, no), and depressive symptoms (yes, no). The 9-item Patient Health Questionnaire (PHQ-9) was used to assess depression, and a threshold of 5 points or more was set to denote the presence of depressive symptoms (Kroenke et al., 2001).

#### 2.4.2 Statistical analysis

SAS V.9.4 (SAS Institute) was used for statistical analysis, with a *p-value* threshold of <0.05, indicating statistical significance.

The baseline characteristics of the participants were summarized using descriptive analyses. For categorical variables (e.g., sex, ethnicity, age groups, education level, work status, economic status, marital status, smoking, drinking, exercise, hypertension, diabetes, stroke, depressive symptoms, BMI groups, WC groups, WHR groups), frequencies and percentages were reported, and the Chi-square test was used to compare differences between participants with and without cognitive impairment. For continuous variables (e.g., BMI, WC, WHR), means and standard deviations were presented, and independent samples *t*-tests were used to compare the mean differences between the two groups.

We utilized log-binomial regression models (Liu, X. H. et al., 2023) to assess the prospective effects of BMI, WC, and WHR on the risk of cognitive impairment among older adults. Model 1 provided crude relative risks (RRs) and 95% confidence intervals (CIs) for cognitive impairment associated with BMI, WC, and WHR. For Model 2, we adjusted for sociodemographic factors such as sex, ethnicity, age, education, work status, economic status and marital status. In Model 3, we additionally adjusted for smoking, drinking, exercise, hypertension, diabetes, stroke, and depressive symptoms. The combined BMI-WC and BMI-WHR grouping strategies were constructed during statistical analysis, rather than predefined in the original study design, in which each composite variable was generated by combining the four BMI categories (underweight, normal, overweight, and obesity) with the respective two-level status (normal vs. central obesity) of WC or WHR, resulting in eight groups for each strategy. Previous epidemiological studies have supported the joint application of general and central obesity indicators, as this approach can more comprehensively reflect body composition and fat distribution characteristics in the elderly (Ortiz et al., 2025). By constructing these composite measures, the relationship between obesity indicators and cognitive impairment was explored further.

Stratified analyses were performed to explore possible variations in the effects of sex and age. We set 70 years as the age cutoff for the stratified analysis, which is clinically meaningful and widely used in research on cognitive impairment (Stessman and Jacobs, 2023; Langa and Levine, 2014). Finally, we conducted sensitivity analyses to assess the robustness of our results. These analyses included excluding participants who exhibited cognitive impairment at the first follow-up, restricting the sample to participants who attended at least one follow-up visit and completed the MMSE on two or more occasions, and defining cognitive impairment as an MMSE score of <24 (a threshold commonly used in Western populations; Tombaugh and McIntyre, 1992).

After adjusting for multiple covariates and combining the exposure categories, all the log-binomial regression models converged successfully.

## 3 Results

### 3.1 Group differences in baseline characteristics

The variations in BMI and WC were notably different between the groups, with significant discrepancies between them. Furthermore, cognitive deficits were linked to a variety of factors, including sex, education, age group, work status, economic status, marital status, smoking, drinking, exercise, hypertension, diabetes, and stroke (Table 1).

### 3.2 Association between BMI, WC, WHR, and cognitive impairment

As shown in Table 2, after further adjustment for other variables in Model 3, the association between underweight and cognitive impairment remained significant (RR = 1.15; 95%CI, 1.05–1.27). Overweight (RR = 0.93; 95%CI, 0.86–1.00) and obesity (RR = 0.91; 95%CI, 0.81–1.01) showed marginally significant inverse associations with cognitive impairment. Compared with individuals with normal WC, those with WC-defined central obesity showed a significant inverse association with cognitive impairment in the initial crude analysis (RR = 0.90; 95%CI, 0.85–0.95). This association remained statistically significant after further adjustment for additional variables in Model 3 (RR = 0.86; 95%CI, 0.81–0.91).

**Table 2:** RR (95%CI) for prospective association between BMI, WC, WHR, and risk of cognitive impairment.

Variables	*N*	Model 1	Model 2	Model 3
BMI groups				
Underweight (<18.5)	422	1.36 (1.22–1.50)	1.17 (1.06–1.29)	1.15 (1.05–1.27)
Normal (18.5–23.9)	5,464	1.00 (reference)	1.00 (reference)	1.00 (reference)
Overweight (24.0–27.9)	1,796	0.92 (0.85–0.99)	0.92 (0.86–0.99)	0.93 (0.86–1.00)
Obesity (≥28.0)	761	0.90 (0.81–1.01)	0.90 (0.81–0.99)	0.91 (0.81–1.01)
WC groups				
Normal	4,081	1.00 (reference)	1.00 (reference)	1.00 (reference)
Central obesity	4,362	0.90 (0.85–0.95)	0.87 (0.83–0.92)	0.86 (0.81–0.91)
WHR groups				
Normal	2,658	1.00 (reference)	1.00 (reference)	1.00 (reference)
Central obesity	5,785	0.97 (0.92–1.04)	0.92 (0.86–0.97)	0.91 (0.86–0.97)

RR = relative risk, CI = confidence interval, BMI = body mass index, WC = waist circumference, WHR = waist-to-hip ratio. Model 1: No adjustments were made for any variables. Model 2: Sex, ethnicity, age, education, work, economic status, and marital status were adjusted. Model 3: Identical covariates from Model 2 and the addition of smoking, drinking, exercise, hypertension, diabetes, stroke, and depressive symptoms.

### 3.3 Association of the combination of BMI and WC/WHR with cognitive impairment

We assessed the combined associations of BMI, WC, and WHR with cognitive impairment by constructing BMI-WC and BMI-WHR composite indices (Table 3). Participants classified as BMI-defined underweight with normal WC/WHR exhibited a positive association with cognitive impairment (RR = 1.12; 95%CI, 1.01–1.23; RR = 1.18; 95%CI, 1.03–1.32). Those with both BMI-defined underweight and WC/WHR-defined central obesity showed no meaningful association (RR = 0.67; 95%CI, 0.35–1.05; RR = 1.06; 95%CI, 0.90–1.22). In addition, participants categorized as BMI-defined overweight with WC/WHR-defined central obesity presented an inverse association with cognitive impairment (RR = 0.88; 95%CI, 0.81–0.95; RR = 0.89; 95%CI, 0.81–0.97). BMI-defined obesity combined with WC/WHR-defined central obesity was also inversely correlated with cognitive impairment (RR = 0.85; 95%CI, 0.75–0.95; RR = 0.88; 95%CI, 0.78–1.00).

**Table 3:** RR (95%CI) for the association of cognitive impairment with the combination of BMI and WC/WHR.

Variables	*N*	Model 1	Model 2	Model 3
BMI + WC				
Underweight+Normal	392	1.36 (1.22–1.51)	1.13 (1.02–1.22)	1.12 (1.01–1.23)
Underweight+Central obesity	30	0.81 (0.42–1.29)	0.66 (0.35–1.04)	0.67 (0.35–1.05)
Normal+Central obesity	2,041	0.93 (0.87–1.01)	0.86 (0.80–0.92)	0.87 (0.81–0.94)
Normal+Normal	3,423	1.00 (reference)	1.00 (reference)	1.00 (reference)
Overweight+Normal	230	0.84 (0.68–1.01)	0.87 (0.71–1.04)	0.88 (0.72–1.05)
Overweight+Central obesity	1,566	0.90 (0.83–0.98)	0.86 (0.80–0.94)	0.88 (0.81–0.95)
Obesity+Normal	36	0.83 (0.46–1.27)	0.94 (0.53–1.43)	0.99 (0.56–1.51)
Obesity+Central obesity	725	0.88 (0.78–0.99)	0.84 (0.75–0.93)	0.85 (0.75–0.95)
BMI + WHR				
Underweight+Normal	244	1.44 (1.24–1.64)	1.18 (1.03–1.34)	1.18 (1.03–1.32)
Underweight+Central obesity	178	1.33 (1.11–1.56)	1.08 (0.92–1.26)	1.06 (0.90–1.22)
Normal+Central obesity	3,459	1.04 (0.97–1.12)	0.97 (0.90–1.04)	0.96 (0.90–1.04)
Normal+Normal	2,005	1.00 (reference)	1.00 (reference)	1.00 (reference)
Overweight+Normal	325	0.99 (0.84–1.16)	1.02 (0.86–1.18)	1.04 (0.88–1.20)
Overweight+Central obesity	1,471	0.93 (0.84–1.02)	0.88 (0.80–0.96)	0.89 (0.81–0.97)
Obesity+Normal	84	0.81 (0.56–1.17)	0.87 (0.60–1.18)	0.91 (0.63–1.24)
Obesity+Central obesity	677	0.94 (0.83–1.06)	0.88 (0.77–0.99)	0.88 (0.78–1.00)

RR = relative risk, CI = confidence interval, BMI = body mass index, WC = waist circumference, WHR = waist-to-hip ratio. Model 1: No adjustments were made for any variables. Model 2: Sex, ethnicity, age, education, work, economic status, and marital status were adjusted. Model 3: Identical covariates from Model 2 and the addition of smoking, drinking, exercise, hypertension, diabetes, stroke, and depressive symptoms.

### 3.4 Stratified analyses of BMI, WC, and WHR associations with cognitive impairment by sex and age

Sex-stratified analyses revealed that after full adjustment in Model 3, WC-defined central obesity exhibited inverse associations with cognitive impairment relative to the normal group, with corresponding RR values of 0.82 (95%CI, 0.75–0.90) and 0.88 (95%CI, 0.82–0.95) in men and women, respectively. WHR-defined central obesity was significantly inversely associated with cognitive impairment in women, whereas no significant association was detected in men. BMI-defined underweight showed a stronger positive association with cognitive impairment among women (RR = 1.17; 95%CI, 1.03–1.32; Supplementary Table S1).

Age-stratified analyses revealed that after adjusting for all covariates in Model 3, WC-defined central obesity was inversely associated with cognitive impairment, with RR = 0.88 (95%CI, 0.80–0.97) in adults aged <70 years and RR = 0.85 (95%CI, 0.79–0.91) in those aged ≥70 years. WHR-defined central obesity had a significant inverse association with cognitive impairment in the ≥70 years group (RR = 0.90; 95%CI, 0.84–0.98), whereas no significant association was found in the <70 years group. Among adults aged >70 years, BMI-defined underweight status was positively associated with cognitive impairment (RR = 1.21; 95%CI, 1.09–1.36; Supplementary Table S1).

The results of the stratified analysis for the association between cognitive impairment and the combination of BMI and WC/WHR are shown in Supplementary Table S2.

### 3.5 Sensitivity analyses support consistency of primary results

The findings of the three sensitivity analyses were consistent with the main results (Table 4).

**Table 4:** RR (95%CI) for the sensitivity analyses of BMI, WC, and WHR in relation to cognitive impairment risk.

Variables	*N*	Model 1	Model 2	Model 3
Sensitivity analysis 1: exclude participants who exhibited cognitive impairment at the first follow-up				
BMI groups				
Underweight (<18.5)	421	1.37 (1.24–1.52)	1.18 (1.07–1.30)	1.16 (1.06–1.28)
Normal (18.5–23.9)	5,429	1.00 (reference)	1.00 (reference)	1.00 (reference)
Overweight (24.0–27.9)	1,786	0.92 (0.85–0.99)	0.92 (0.86–0.99)	0.93 (0.87–1.00)
Obesity (≥28.0)	761	0.92 (0.82–1.02)	0.91 (0.82–1.01)	0.92 (0.83–1.02)
WC groups				
Normal	4,058	1.00 (reference)	1.00 (reference)	1.00 (reference)
Central obesity	4,339	0.89 (0.84–0.95)	0.85 (0.80–0.90)	0.86 (0.81–0.91)
WHR groups				
Normal	5,750	1.00 (reference)	1.00 (reference)	1.00 (reference)
Central obesity	2,647	0.97 (0.92–1.04)	0.92 (0.86–0.97)	0.91 (0.86–0.97)
Sensitivity analysis 2: in participants who completed at least two MMSE assessments during follow-up				
BMI groups				
Underweight (<18.5)	386	1.35 (1.21–1.51)	1.17 (1.06–1.30)	1.16 (1.05–1.29)
Normal (18.5–23.9)	4,974	1.00 (reference)	1.00 (reference)	1.00 (reference)
Overweight (24.0–27.9)	1,679	0.93 (0.86–1.00)	0.93 (0.86–1.00)	0.94 (0.87–1.01)
Obesity (≥28.0)	712	0.90 (0.80–1.00)	0.89 (0.80–0.99)	0.90 (0.81–1.01)
WC groups				
Normal	3,671	1.00 (reference)	1.00 (reference)	1.00 (reference)
Central obesity	4,080	0.88 (0.83–0.94)	0.84 (0.79–0.89)	0.85 (0.80–0.90)
WHR groups				
Normal	2,411	1.00 (reference)	1.00 (reference)	1.00 (reference)
Central obesity	5,340	0.97 (0.91–1.03)	0.91 (0.86–0.97)	0.91 (0.85–0.97)
Sensitivity analysis 3: defining cognitive impairment as MMSE score <24				
BMI groups				
Underweight (<18.5)	422	1.35 (1.23–1.49)	1.12 (1.03–1.21)	1.11 (1.03–1.20)
Normal (18.5–23.9)	5,464	1.00 (reference)	1.00 (reference)	1.00 (reference)
Overweight (24.0–27.9)	1,796	0.91 (0.85–0.98)	0.94 (0.88–1.00)	0.94 (0.89–1.00)
Obesity (≥28.0)	761	0.92 (0.84–1.02)	0.93 (0.85–1.01)	0.94 (0.86–1.02)
WC groups				
Normal	4,081	1.00 (reference)	1.00 (reference)	1.00 (reference)
Central obesity	4,362	0.90 (0.86–0.95)	0.87 (0.82–0.91)	0.89 (0.84–0.93)
WHR groups				
Normal	2,658	1.00 (reference)	1.00 (reference)	1.00 (reference)
Central obesity	5,785	0.98 (0.93–1.04)	0.91 (0.86–0.97)	0.91 (0.86–0.96)

RR = relative risk, CI = confidence interval, BMI = body mass index, WC = waist circumference, WHR = waist-to-hip ratio. Model 1: No adjustments were made for any variables. Model 2: Sex, ethnicity, age, education, work, economic status, and marital status were adjusted. Model 3: Identical covariates from Model 2 and the addition of smoking, drinking, exercise, hypertension, diabetes, stroke, and depressive symptoms.

## 4 Discussion

In this 6-year cohort study, we found that older adults classified as underweight according to the BMI standards exhibited a notably stronger positive association with cognitive impairment. Moreover, central obesity, as measured by WC or WHR, was inversely associated with cognitive impairment. The associations were particularly pronounced in participants aged ≥70 years and in women. Individuals with BMI-defined underweight but WC/WHR-defined central obesity showed no association with cognitive impairment. Participants with both BMI-defined overweight/obesity and WC/WHR-defined central obesity had a significantly stronger inverse association with cognitive impairment.

Previous research findings on the relationships between BMI, WC, WHR, and cognitive impairment in older adults have been inconsistent. Some studies suggest that higher values of these three indicators increase the risk of cognitive impairment, whereas others have indicated an inverse association. Multiple studies have found that BMI–defined underweight is associated with an increased risk of cognitive impairment (Zhang et al., 2021; Zhang et al., 2025; Borda et al., 2021; Wang et al., 2017). Zhu et al. found that (Zhu et al., 2024) female underweight (BMI < 18.5 kg/m^2^) was significantly associated with cognitive impairment (OR = 1.32; 95%CI, 1.20–1.46), whereas this association was not significant among males. Another study involving 9,218 individuals aged 80 and older found that (Chen et al., 2022) underweight status maintains a significant positive association with cognitive impairment, particularly among women. These findings are consistent with the results of this study: BMI-defined underweight presents a significant positive association with cognitive impairment in Chinese older adults. However, a few studies have yielded results that differ from those of the present study’s. For instance, Priya et al. (Balasubramanian, 2021) found that being overweight or obese significantly increased the risk of cognitive impairment in older adults. Quaye et al. (2023) found that obesity is associated with cognitive decline among older Americans, and this correlation differs across racial groups. A study involving 17,352 community-dwelling older adults found that Yi et al. (2024) both underweight and obesity increased the risk of cognitive impairment in older adults. This discrepancy may stem from the specific context of the study population and the methodologies used. Our study population consisted of older adults from the Zhejiang Province in China. Their genetic predispositions, lifestyle habits, and traditional dietary patterns, characterized by a diet rich in fish and plant-based foods with potentially low saturated fat intake, may result in fundamentally different associations between obesity levels and health outcomes than those in other regions worldwide. Another finding of this study was that WC/WHR-defined central obesity was inversely associated with cognitive impairment in older adults. This conclusion differs from previous findings that regarded central obesity as having a positive association with cognitive impairment (Smith et al., 2022; Zhou et al., 2011). However, such contradictory findings are not uncommon in studies on obesity and health among older adults. Liu X et al. explicitly demonstrated that (Liu et al., 2021) a higher WHR may be associated with better cognitive function. However, this association was observed exclusively in women aged ≥70 years. Second, a cross-sectional study by Liang et al. similarly found (Liang et al., 2024) a significant inverse association between WC and the risk of cognitive impairment (OR = 0.93; 95%CI, 0.88–0.98). Furthermore, a study of older adults with an average age exceeding 73 years demonstrated that (Luchsinger et al., 2013) a larger waist circumference was associated with a slower rate of cognitive decline among Americans.

Multiple biological mechanisms may account for the observed results. First, low body weight and sarcopenia often coexist. Reduced muscular irisin secretion weakens neuroprotection and increases cognitive decline risk (Nishiguchi et al., 2016; Hu, F. et al., 2021; Hu, M. et al., 2021). Second, underweight status is linked to higher systemic inflammation. IL-6, TNF-*α* and other pro-inflammatory cytokines cross the blood–brain barrier to induce neuroinflammation and oxidative injury, and this effect is more pronounced in the elderly (Estrada and Contreras, 2019; Di Benedetto and Müller, 2019). Third, subcutaneous fat secretes adiponectin, which exerts anti-inflammatory and insulin-sensitizing effects to protect the brain (Bloemer et al., 2018). Women have more subcutaneous fat, and postmenopausal estrogen decline leads to abdominal fat gain and fat redistribution. Subcutaneous fat is metabolically milder than visceral fat and serves as a safe energy reserve (Kuryłowicz, 2023; UT Southwestern Medical Center, 2025). Since this study did not measure the aforementioned indicators, any explanation of the biological mechanisms described above remain speculative.

In addition, the findings of this study may be related to the “obesity paradox.” The core feature of this paradox lies in the observation that, among the elderly population, higher body weight or obesity-related indicators are sometimes positively correlated with lower mortality rates and better health outcomes (Lv et al., 2024; Gavriilidou et al., 2024). This suggests that in older adults, adequate fat reserves may signify stronger energy reserves and survival advantages (Zaslavsky et al., 2017). However, some studies have cast doubt on the validity of the “obesity paradox” (Zhang et al., 2024; Niedziela et al., 2014). Although our findings regarding the association between central obesity phenotypes and cognitive impairment are consistent with the “obesity paradox” hypothesis, the present study lacked standardized assessments of sarcopenia, frailty, detailed dietary intake, and precise body composition, leaving the effects of nutritional reserves and body composition unclear. These unmeasured factors may introduce potential residual confounding, thereby limiting the mechanistic interpretation of the observed paradox.

Furthermore, multiple studies have indicated that metabolically healthy obese individuals (those who are obese but have normal key metabolic indicators) do not exhibit a higher risk of cognitive decline than non–obese individuals (Ma et al., 2019; Lee et al., 2019; Kastorini et al., 2012). Given that this study did not collect data on metabolic markers such as blood glucose and lipid levels, it remains unclear whether the study population included a significant proportion of individuals with abdominal obesity but good metabolic health status. Such individuals typically exhibit favorable metabolic profiles, including normal glucose tolerance, lipid levels, and inflammation levels. These characteristics may mitigate the potential risks associated with simple abdominal obesity, leading to the inverse association observed in the study population (Lazzaris Coelho et al., 2026).

Beyond metabolic heterogeneity, the accurate evaluation of visceral fat distribution and body composition relies on precise, quantitative techniques. The inherent measurement limitations of BMI, WC, and WHR may impede the accurate assessment of visceral fat deposition and individual body composition in the elderly population. Emerging evidence suggests that (Xu et al., 2025) conventional anthropometric indicators do not fully reflect the dynamic metabolic remodeling processes involved in the pathophysiology of obesity. Metabolomic-based metabolic phenotyping has revealed that metabolic status, rather than fat mass, is the primary determinant of disease prognosis. Therefore, metabolic phenotyping has greater clinical value than assessments based solely on anthropometric measures.

The strengths of this study are as follows: (1) The longitudinal study design, coupled with an adequate follow-up period, enabled the examination of the temporal relationships between exposure and outcome. (2) The use of a large community-based sample of older adults enhances the generalizability of our relatively robust findings to the broader elderly population in China. (3) We evaluated the combined effects of BMI and WC/WHR on the risk of cognitive impairment, which has not been previously studied.

The limitations and methodological issues of this study are as follows: (1) The lack of circulating metabolic markers (e.g., blood glucose and lipids) hinders the differentiation between metabolically healthy and unhealthy obesity, while the inability to precisely distinguish body components, such as visceral and subcutaneous fat, limits the in-depth analysis of the effects of fat distribution. (2) Potential attrition, survival bias and competing mortality risk cannot be fully ruled out. Participants may be lost to follow-up due to death, relocation or loss of contact, and such individuals tend to differ systematically from those who remained in the cohort. Notably, older adults with poor baseline health and a higher risk of cognitive decline were more likely to drop out or die prematurely. Since premature mortality acts as a typical competing risk, these participants passed away before cognitive outcomes could be assessed. Consequently, the final analytical sample only included relatively healthy survivors, which may introduce bias to our results. (3) Additionally, we failed to account for preclinical disease-related body changes. Accumulated evidence indicates that involuntary weight loss often precedes cognitive decline and dementia among older adults by several years. In this context, low BMI and reduced body fat mass are more likely to be indicators of preclinical cognitive disorders rather than independent causal risk factors (Wu et al., 2024; Kivimäki et al., 2018). (4) Furthermore, reverse causation remains a potential concern. Although we performed a sensitivity analysis by excluding participants who developed cognitive impairment in the first follow-up wave to reduce such interference, reverse causation could not be completely eliminated, which is another inherent limitation of the current analysis.

Future studies should integrate metabolic biomarkers and detailed body composition assessments (including visceral fat and sarcopenia), potentially combined with AI-assisted risk stratification. It should also translate population-based epidemiological observations into technology-driven precision risk assessment tools and facilitate their translation into actionable public health strategies and clinical practice (Ning et al., 2026). This approach can effectively integrate heterogeneous clinical and omics data to reveal complex nonlinear relationships, thereby helping to elucidate the intricate associations between obesity phenotypes and cognitive impairment in older adults (Veneziani et al., 2024). Simultaneously, more precise measurement tools should be used in conjunction with various neuropsychological scales to reduce classification bias, thereby laying the groundwork for developing targeted prevention strategies for the same.

## 5 Conclusion

In summary, this study revealed a complex association between body composition and cognitive impairment in older Chinese adults. The findings indicate that BMI-defined underweight shows a clear positive association with cognitive impairment, whereas WC-defined- and WHR-defined central obesity shows an inverse association. These associations were particularly pronounced in individuals aged ≥70 years and in women.

This finding suggests that the traditional paradigm of viewing obesity as a health risk may not apply to all older adults with obesity. Nutritional reserves, and metabolic health also need to be highlighted in the elderly population. Given that the findings of this study represent observational associations, further validation is needed through longitudinal studies that incorporate body composition measurements, metabolic biomarkers, and repeated cognitive assessments.

## Data Availability

The raw data supporting the conclusions of this article will be made available by the authors, without undue reservation.
