# Supplementary material for: A 6-year cohort study of the associations of body mass index, waist circumference, and waist-hip ratio with cognitive impairment in Chinese elderly
Source: Front Psychol. 2026 Jul 3;17:1833217. doi: 10.3389/fpsyg.2026.1833217 (PMC13376249; doi:10.3389/fpsyg.2026.1833217)
Supplement: Supplementary file 1 [file Table_1.docx]

**Table S1^a^ RR (95%CI) for** **prospective association between BMI, WC, WHR and risk of cognitive impairment**

| **Variable** | **Sex** | | **Age groups** | |
| --- | --- | --- | --- | --- |
|  | **Men** | **Women** | **<70** | **≥70** |
| **BMI** |  |  |  |  |
| Underweight(＜18.5) | 1.16 (0.99-1.36) | 1.17 (1.03-1.32) | 1.20 (0.99-1.46) | 1.21 (1.09-1.36) |
| Normal(18.5-23.9) | 1.00 (reference) | 1.00 (reference) | 1.00 (reference) | 1.00 (reference) |
| Overweight(24-27.9) | 0.87 (0.77-0.98) | 0.97 (0.89-1.06) | 0.93 (0.83-1.05) | 0.91 (0.83-1.00) |
| Obesity(≥28) | 0.84 (0.69-1.03) | 0.93 (0.82-1.06) | 0.85 (0.71-1.01) | 0.91 (0.80-1.04) |
| **WC** |  |  |  |  |
| Normal | 1.00 (reference) | 1.00 (reference) | 1.00 (reference) | 1.00 (reference) |
| Central obesity | 0.82 (0.75-0.90) | 0.88 (0.82-0.95) | 0.88 (0.80-0.97) | 0.85 (0.79-0.91) |
| **WHR** |  |  |  |  |
| Normal | 1.00 (reference) | 1.00 (reference) | 1.00 (reference) | 1.00 (reference) |
| Central obesity | 0.91 (0.84-1.00) | 0.91 (0.84-0.99) | 0.96 (0.87-1.06) | 0.90 (0.84-0.98) |

Abbreviations: RR = Relative Risk, CI = Confidence Interval, BMI = body mass index, WC = waist circumference, WHR = waist-to-hip ratio.

Model 1: No adjustments were made for any variables.

Model 2: Ethnicity, education, work, economic status, and marital status were adjusted.

Model 3: Identical covariates from Model 2 and the addition of smoking, drinking, exercise, hypertension, diabetes, stroke, and depressive symptoms.

a: This table only presents the results of Model 3.

| **Variable** |  | **Men** | **Women** | **<70** | **≥70** |
| --- | --- | --- | --- | --- | --- |
| **BMI+WC** |  |  |  |  |  |
|  | Underweight+Central obesity | 0.74 (0.21-1.44) | 0.63 (0.32-1.23) | 0.83 (0.31-2.16) | 0.66 (0.34-1.27) |
|  | Underweight+Normal | 1.14 (0.96-1.32) | 1.15 (1.01-1.32) | 1.18 (0.96-1.44) | 1.19 (1.06-1.34) |
|  | Normal+Central obesity | 0.86 (0.76-0.97) | 0.88 (0.81-0.97) | 0.90 (0.79-1.01) | 0.87 (0.79-0.95) |
|  | Normal+Normal | 1.00 (reference) | 1.00 (reference) | 1.00 (reference) | 1.00 (reference) |
|  | Overweight+Central obesity | 0.79 (0.69-0.91) | 0.93 (0.84-1.03) | 0.90 (0.79-1.03) | 0.86 (0.77-0.96) |
|  | Overweight+Normal | 1.10 (0.78-1.25) | 0.74 (0.52-1.04) | 0.85 (0.63-1.14) | 0.86 (0.67-1.11) |
|  | Obesity+Central obesity | 0.81 (0.65-1.00) | 0.86 (0.75-0.99) | 0.81 (0.67-0.97) | 0.85 (0.74-0.99) |
|  | Obesity+Normal | 0.67 (0.26-1.27) | 1.69 (0.96-2.95) | 0.84 (0.35-2.00) | 0.99 (0.55-1.76) |
| **BMI+WHR** |  |  |  |  |  |
|  | Underweight+Central obesity | 1.18 (0.92-1.50) | 1.02 (0.84-1.25) | 1.12 (0.80-1.57) | 1.13 (0.94-1.36) |
|  | Underweight+Normal | 1.13 (0.92-1.38) | 1.23 (1.05-1.44) | 1.24 (0.97-1.59) | 1.23 (1.07-1.42) |
|  | Normal+Central obesity | 0.98 (0.88-1.08) | 0.97 (0.88-1.07) | 1.00 (0.88-1.12) | 0.97 (0.88-1.06) |
|  | Normal+Normal | 1.00 (reference) | 1.00 (reference) | 1.00 (reference) | 1.00 (reference) |
|  | Overweight+Central obesity | 0.79 (0.67-0.92) | 0.95 (0.85-1.07) | 0.93 (0.80-1.07) | 0.87 (0.77-0.98) |
|  | Overweight+Normal | 1.05 (0.87-1.28) | 1.00 (0.79-1.26) | 1.01 (0.97-1.28) | 1.04 (0.86-1.27) |
|  | Obesity+Central obesity | 0.84 (0.67-1.04) | 0.92 (0.80-1.06) | 0.88 (0.73-1.07) | 0.87 (0.75-1.02) |
|  | Obesity+Normal | 0.83 (0.49-1.39) | 1.00 (0.68-1.48) | 0.57 (0.28-1.13) | 1.10 (0.78-1.57) |

**Table S2^b^ RR (95%CI) for the association of cognitive impairment with the combination of BMI and WC/WHR**

Abbreviations: RR = Relative Risk, CI = Confidence Interval, BMI = body mass index, WC = waist circumference, WHR = waist-to-hip ratio.

Model 1: No adjustments were made for any variables.

Model 2: Ethnicity, education, work, economic status, and marital status were adjusted.

Model 3: Identical covariates from Model 2 and the addition of smoking, drinking, exercise, hypertension, diabetes, stroke, and depressive symptoms.

b:This table only presents the results of Model 3.
